# Davis flap: the glory still present

**DOI:** 10.3205/iprs000093

**Published:** 2016-05-04

**Authors:** Ahmed Hassan El-Sabbagh

**Affiliations:** 1 Plastic Surgery Center, Faculty of Medicine, Mansoura University, Mansoura, Egypt

**Keywords:** ear, upper third defect, Davis flap

## Abstract

**Background:** Upper third defects of the ear are too large to be closed primarily without distorting the auricle. Full thickness defects can be reconstructed with local flaps. In this article, Davis flap was used to fill the upper third defects of the ear with some modifications.

**Patients and methods:** Eight patients underwent reconstruction of full thickness auricular defects with Davis flaps from July 2012 to December 2014. The posterior surface of the flap and the raw area of conchal area were covered by full thickness graft taken from posterior surface of ear.

**Results:** All flaps survived. No congestion was noted. The donor sites and skin grafts healed uneventfully.

**Conclusion:** Davis flap is a simple and reproducible tool for reconstruction of upper third of ear.

## Zusammenfassung

**Hintergrund:** Ohrmuscheldefekte im oberen Drittel sind zu groß für einen primären Nahtverschluss ohne Beeinträchtigung der Muschelkontur.

In der vorliegenden Arbeit wurde der Konchatranspositionslappen nach Davis (Davis-Flap) mit gewissen Modifikationen zur Defektauffüllung im oberen Muscheldrittel verwendet.

**Patienten und Methoden:** Acht Patienten nach ausgedehnten oberen Ohrmuscheldefekten wurden in der Zeit von Juli 2012 bis Dezember 2014 rekonstruiert. Hierbei wurde die Rückfläche des transponierten Konchalappens und Entnahmestelle durch Volltransplantat von der Rückseite der Ohrmuschel gedeckt.

**Ergebnisse:** Alle Transpositionslappen überlebten. Schwellungen wurden nicht beobachtet. Die Entnahmestellen heilten sämtlich problemlos.

**Schlussfolgerung:** Der Konchatranspositionslappen nach Davis ist ein einfaches und verlässliches Tool für die Rekonstruktion größerer Ohrmuscheldefekte im oberen Drittel.

## Introduction

Due to the unique anatomical parts of the ear, a cosmetically acceptable reconstruction for ear defects stills a big issue [1], [2]. In addition to limitation of donor tissue, reconstruction of posttraumatic ear deformities abused most of the basic principles in plastic surgery.

A number of methods have been described for upper third ear defects [3], [4]. This may reflect the lack of one uniformly accepted method of reconstruction.

In this study, Davis flap was used to reconstruct the upper third of the ear. All the defects were full thickness and full thickness grafts were used to cover the conchal defect.

## Patients and methods

Eight patients underwent reconstruction of upper third auricular defects with Davis flaps from November 2012 to February 2016. Defects were caused by human bites (n=4), motor car accident (n=3), and cutting by knife (n=1). Informed consent was obtained from all patients involved in the study. Separate consent for photography was taken from all cases involved in the study.

## Technique

**Figure 1:**
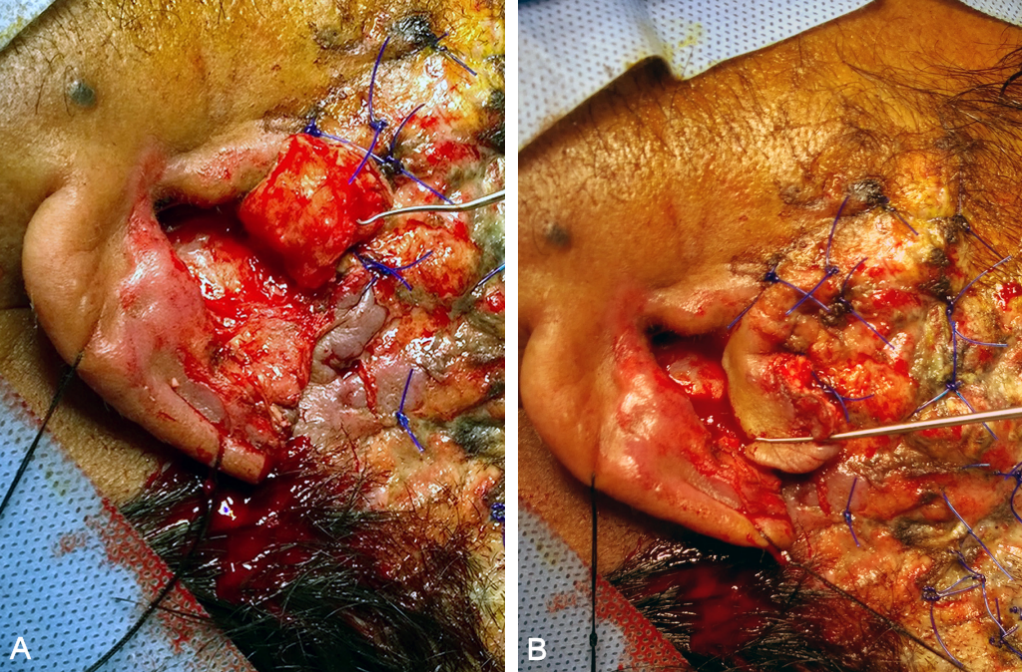
Technique for Davis flap. A) Undersurface of the flap. B) Transposition of the flap.

Highlighted by Davis’ description [5], the flap was based on the root of the helix (Figure 1 (Fig. 1)). It contained the anterior skin and the underlying conchal cartilage. The posterior skin was left intact. Then, the flap was rotated upwards to fill the defect in the upper third of the ear. The raw surface of the conchal area was closed with a full thickness skin graft taken from posterior auricular surface. The donor site of full thickness graft was closed primarily.

## Results

**Figure 2:**
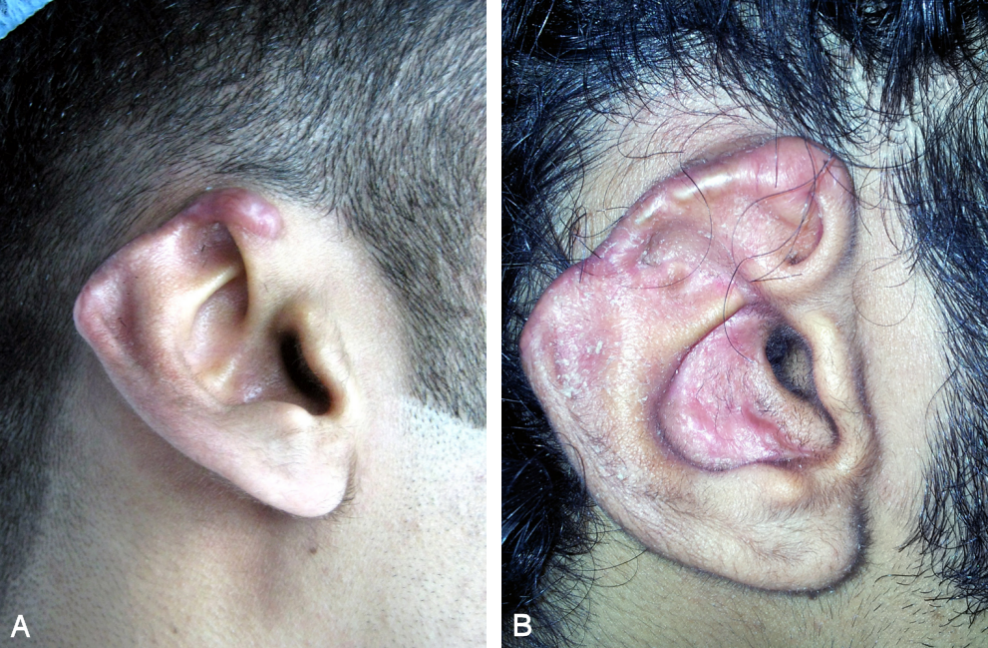
Loss of upper third of right ear after human bite injury. A) Loss of upper third after debridement. B) 6 month postoperative.

**Figure 3:**
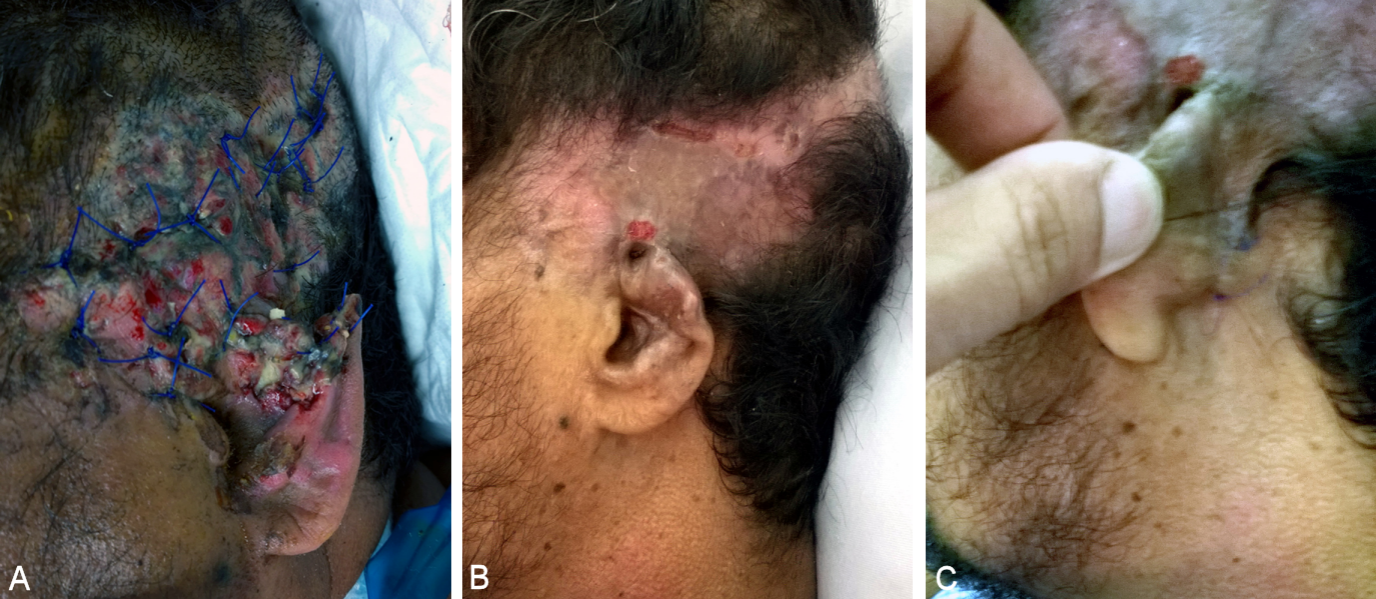
Loss of upper third of left ear after motor car accident. A) Loss of upper third. B) Complete healing of the flap (2 months postoperative). C) Complete healing of graft donor site with preservation of postauricular sulcus (2 months postoperative).

**Figure 4:**
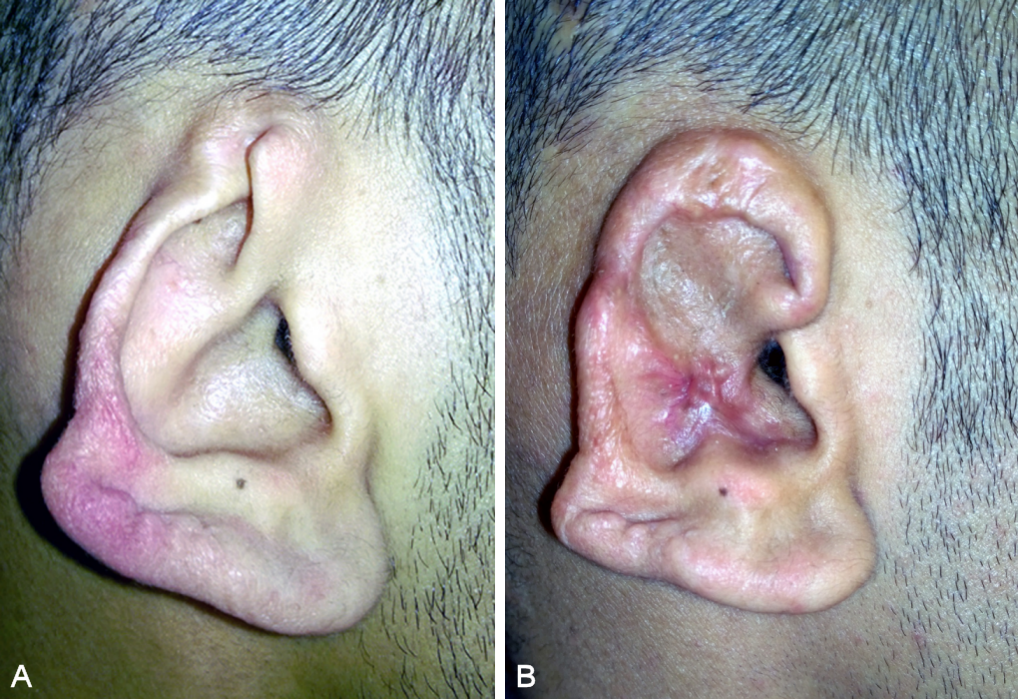
Loss of upper third of left ear by knife. A) Loss of upper third. B) Complete healing of the flap after 3 months.

**Figure 5:**
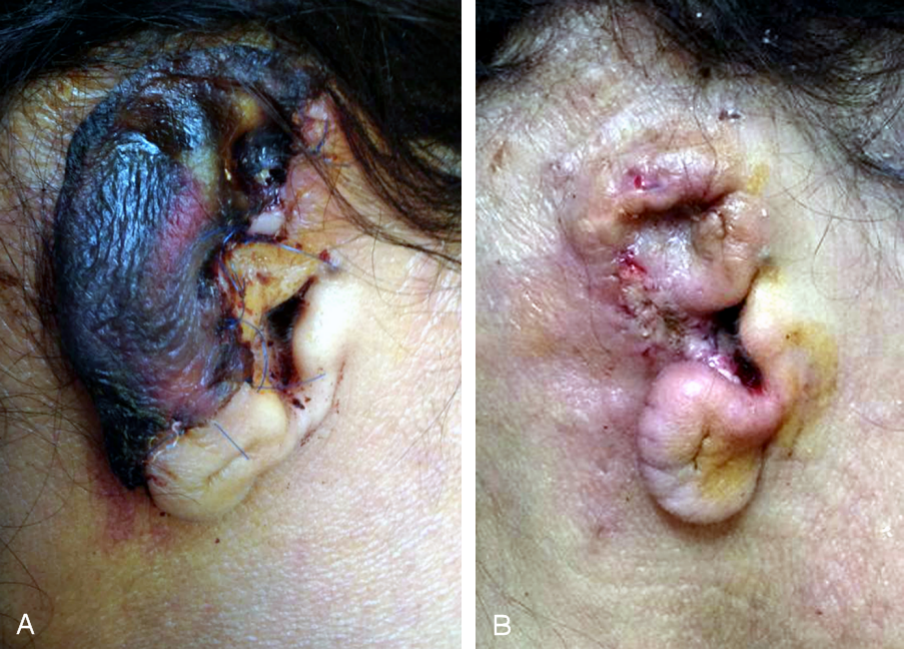
Ischemic right ear due to human bite. A) Ischemic right ear. B) Davis flap for upper third defect.

Patients were seven men and one woman. Their age ranged from 19 years to 57 years. All flaps survived with good healing (Figure 2 (Fig. 2), Figure 3 (Fig. 3), Figure 4 (Fig. 4), Figure 5 (Fig. 5)). No congestion was noted in the series. The suture lines were well hidden, and the aesthetic results were satisfactory. There was no auricular deformation. All grafts survived completely and the donor sites showed good healing. There was no keloid nor hypertrophic scar formations in all cases.

## Discussion

The ear is an aesthetic structure of intricate cartilage folds covered by skin. Loss of an ear, in whole or in part, can cause psychological distress out of proportion to its size and appearance. Some cases had developed serious behavior abnormalities and mood deviations [1].

Reconstruction of partial amputations of the auricle is different from complete reconstruction. Partial amputation of the auricle was a field for multiple publications. These publications gave the priority for reconstruction by local flaps [2], [3], [4], [5], [6], [7].

Needless to say, repair of an ear deformity needs both accurate analysis of the defect and a planning for the reconstruction [8]. Brent [9] had described several solutions for repair of upper-third defects. The decision was dependent on the size of the defect and how much available tissue remains for the reconstruction.

For small helical rim defects, Antia-Buch chondrocutaneous advancement flaps are used. Larger defects that miss the cartilaginous support are best treated with preauricular banner flaps combined with auricular cartilage grafts at the helical rim [10].

In major defects of the upper third of the ear, the reconstructive options depend on whether available skin is sufficient for a local flap or not. Contralateral cartilage graft can be used, if postauricular skin flap is available. If there is not enough skin, a compound pedicled flap is used for reconstruction [11].

In the 1970s, two chondrocutaneous flaps were described by Davis and Orticochea. Davis flap was based on the root of the helix and was transposed to the marginal defect, whereas the Orticochea flap was based on the caudal part of the helix [5], [9].

In this article, the Davis flap was used to reconstruct the upper third of ear and the conchal raw area was covered by a full thickness graft taken from post auricular area.

Yotsuyanagi designed a reconstruction that consists of three flaps [10]. A chondrocutaneous flap is raised from the conchal bowl and is advanced into the defect to provide cartilage support and anterior skin coverage. A postauricular flap is transposed to provide posterior skin coverage of the wound. The defect that develops after elevating the postauricular subcutaneous pedicled flap is covered with a full-thickness skin graft [10].

Also, Yoshimura and his colleagues described a combined postauricular skin flap and a mastoid fascial flap [11]. The two flaps were then used to sandwich a fabricated costal cartilage framework. The donor area for the mastoid fascial flap was skin grafted. The difference of this technique from that presented by Yotsuyanagi et al. was in using free rib cartilage graft instead of chondrocutaneous flap [10].

In addition, Dagregorio and Darsonval elevated a chondrocutaneous flap for upper and middle-third defects involving the helical rim. It is an axial flap based on the posterior auricular artery and the auricular branch of the superficial temporal artery. Actually, it is a convenient flap in comparison to multi-staged procedures involving costal cartilage grafts [12].

Anatomically, the concha is a hollow bowl-like portion of the external ear that leads to the ear canal. Its volume is approximately 4 cm and the cavity is partially divided by the crus helix and the cymba which is connected to the fossa [13].

Raw area of the concha could be covered by tunneling the mastoid fascia through the ear [14]. However, there is possibility of leaving the defect of the conchal area, without skin coverage and therefore allowing secondary healing. The raw area will heal secondarily without ear deformity because of the surrounding cartilage [15], [16]. In this paper, full thickness graft was harvested from the postauricular area from the same ear with excellent results. Tie over dressing over the conchal area was removed after ten days.

Advantages of Davis conchal flap includes that it is a single-stage operation, simple, easy to do with a good color and texture match. In addition, there is a concealed donor site. A disadvantage of this flap is that the conchal area is made of two layers of skin that are thin and may contract [16].

Although not frequent, the formation of keloid scars in the auricular region is a known complication after ear piercing and surgery for bat ears in susceptible individuals [17], [18], [19], [20]. Fortunately, none of our cases showed keloid formation or hypertrophic scar.

A limitation of this study was the small number of cases. However, this flap did not need a learning curve or was not accompanied by major complications that necessitate large number of cases to evaluate.

Sir Winston Churchill said that the longer you look back the further you can look forward. Although Davis flap was described in the 70s, the flap still acts as a working horse tool for building up the upper third of ear.

In summary, Davis technique is an ideal flap for reconstruction of upper third of the ear. It could be an alternative to more complicated procedures involving rib harvesting.

## Conclusion

The ideal reconstructive procedure should be simple, one stage and replace like with like for ensuring a good aesthetic outcome. These criteria are fulfilled with Davis flap technique.

## Notes

### Competing interests

The author declares that he has no competing interests.

### Ethical standards

This study has been performed in accordance with the ethical standards set forth in the 1964 Declaration of Helsinki and its later amendments. Informed consent was obtained from all parents responsible for participants included in the study.
